# Low-dose naltrexone as an adjunctive treatment for major depressive disorder: findings from a randomized, double-blind, placebo-controlled hybrid parallel-arm study

**DOI:** 10.3389/fphar.2026.1767654

**Published:** 2026-03-06

**Authors:** Ben D. Moloney, Anna Forsyth, Rachael L. Sumner, Stephanie C. Glover, Nicholas R. Hoeh, Frederick Sundram, Alana Cavadino, Suresh Muthukumaraswamy, Joanne C. Lin

**Affiliations:** 1 School of Pharmacy, Faculty of Medical and Health Sciences, University of Auckland, Auckland, New Zealand; 2 Department of Biomedicine and Medical Diagnostics, Auckland University of Technology, Auckland, New Zealand; 3 Department of Psychological Medicine, School of Medicine, Faculty of Medical and Health Sciences, University of Auckland, Auckland, New Zealand; 4 Department of Epidemiology and Biostatistics, School of Population Health, Faculty of Medical and Health Sciences, University of Auckland, Auckland, New Zealand

**Keywords:** C-reactive protein, inflammation, major depressive disorder, naltrexone, rct

## Abstract

**Introduction:**

Major depressive disorder (MDD) is a leading cause of global disability. Current treatments are limited by poor efficacy in approximately one-third of patients. Neuroinflammation may be an underlying mechanism of MDD and represents a novel target for pharmacological therapy. This study aimed to investigate the effects of a putative centrally acting anti-inflammatory agent, low-dose naltrexone (LDN), in MDD.

**Methods:**

Patients with MDD experiencing moderate depressive symptoms and receiving antidepressant treatment were randomized to receive 12 weeks of LDN (up to 4.5 mg per day) or 12 weeks of inactive placebo. The primary outcome measure was the Montgomery-Asberg Depression Rating Scale (MADRS) at 12 weeks, analyzed using a linear mixed-effects model adjusted for baseline.

**Results:**

Thirty-seven patients were randomized. At 12 weeks, MADRS scores (M ± SD) were reduced by 10.5 ± 5.6 in the LDN group and 9.8 ± 5.9 placebo group; with no difference between groups (p = 0.97). LDN did not affect high-sensitivity C-reactive protein (hsCRP) levels or exploratory measures of depression, behavioral activation, quality of life, sickness symptoms and mood. There was no evidence that baseline hsCRP modified the effect of LDN on MADRS score.

**Discussion:**

Adjunctive LDN does not appear to alter depressive symptoms in moderate MDD. Larger studies are warranted to evaluate LDN in a population with a higher likelihood of neuroinflammatory pathology, such as those with severe, treatment-resistant MDD or comorbid inflammatory conditions. Future studies should utilize stratification tools that are more sensitive and specific to neuroinflammation than hsCRP.

**Clinical Trial Registration:**

https://www.anzctr.org.au/Trial/Registration/TrialReview.aspx?id=383741&isReview=true, identifier [ACTRN12622000881730].

## 1 Introduction

Major depressive disorder (MDD) is one of the most common psychiatric illnesses worldwide, affecting over 5% of the world’s adult population (World Health Organization, 2025). MDD is characterized by a significant and persistent change in an individual’s emotions, activity and cognition (Malhi et al., 2021). The early treatment of MDD relies heavily on the use of antidepressant medication; however, over 60% of patients will find their first trial of an antidepressant ineffective (Warden et al., 2007). While the mechanisms underlying MDD remain complex and poorly understood, neuroinflammation is being increasingly investigated for its role.

Evidence suggests that inflammatory mechanisms may underlie up to one-third of cases of depression (Bullmore, 2018). MDD has been associated with heightened inflammatory markers like C-reactive protein (CRP) (Raison et al., 2006). MDD symptomatology overlaps with sickness-related symptoms, such as poor appetite, fatigue, lethargy and anhedonia, which are the body’s normal physiological response to increased pro-inflammatory cytokines during a period of infection (Fu et al., 2010). MDD is also commonly comorbid with conditions that involve neuroinflammation, such as Parkinson’s disease and stroke (Price et al., 2011). Evidence points towards a specific role of neuroinflammation in MDD (Enache et al., 2019); however, there are currently no accepted treatments for MDD that target neuroinflammation.

Anti-inflammatory agents have been the subject of several trials in MDD, yet they have yielded mixed results. Heterogeneity in cohort characteristics, treatment mechanism of action, dosing regimen, and outcome measures has made it challenging to draw mechanistically informed conclusions regarding the efficacy of anti-inflammatories in MDD (Simon et al., 2023). The strongest evidence of antidepressant efficacy exists for blood-brain barrier-permeable anti-inflammatories like celecoxib and minocycline (Simon et al., 2023); however, long-term use of these drugs is associated with cardiovascular and gastrointestinal adverse effects, which may paradoxically heighten neuroinflammation (Thompson et al., 2020; Solomon et al., 2008; Eyre et al., 2015), limiting their utility in practice.

Naltrexone, an atypical opioid receptor antagonist, has purported anti-inflammatory and immunomodulatory effects in the central nervous system (Younger et al., 2014). Commonly known to be indicated for opioid and alcohol addiction, when used in low doses of 0.5–5 mg (1/100 th to 1/10 th of the dose for addiction), naltrexone has been shown to reduce disease severity in conditions with neuroinflammatory pathology such as multiple sclerosis, fibromyalgia, and chronic pain (Patten et al., 2018). Studies have identified off-target antagonistic effects of naltrexone on toll-like receptor 4 (TLR-4) on microglia (Cant et al., 2017; Grace et al., 2015; Hutchinson et al., 2008). TLR-4 inhibition reduces microglial activation, decreasing the production of CNS cytokines interleukin (IL)-1β, IL-6 and tumor necrosis factor (TNF)-α which elicit sickness behaviors (Cant et al., 2017; Grace et al., 2015; Hutchinson et al., 2008; Kucic et al., 2021). Additionally, at low doses, naltrexone intermittently blocks opioid receptors, upregulating endorphinergic and enkephalinergic signaling and reducing neuroimmune cell proliferation via the opioid growth factor axis (Donahue et al., 2011; Hammer et al., 2016).

A small pilot trial of low-dose naltrexone (LDN) in 12 patients with MDD refractory to pro-dopaminergic antidepressant regimens showed that those receiving LDN experienced a greater reduction in depressive symptoms at 3 weeks compared to placebo (Mischoulon et al., 2017). A 12 week course of LDN also improved mood and general satisfaction with life in a small randomized controlled crossover trial in 31 women with fibromyalgia (Younger et al., 2013). LDN has been shown to reduce serum cytokines and increase opioidergic signaling in fibromyalgia and multiple sclerosis, respectively (Gironi et al., 2008; Ludwig et al., 2017; Parkitny and Younger, 2017). Therefore, a larger clinical trial of LDN in MDD, focusing on its anti-inflammatory mechanisms, would help elucidate the effects of LDN and clarify its potential utility as a treatment for MDD.

We conducted a 12 week randomized, double-blind, placebo-controlled, parallel-arm study of LDN (4.5 mg per day) as an adjunctive treatment for MDD. Stratification of our sample by high-sensitivity CRP (hsCRP) prior to randomization was employed to explore whether treatment effects varied according to inflammatory status. We hypothesized that adjunctive LDN would demonstrate superior treatment efficacy in MDD compared to placebo, particularly in individuals with elevated hsCRP.

## 2 Materials and methods

### 2.1 Study design

The current study was a double-blind, parallel-arm, hybrid randomized controlled trial (RCT) designed to assess the superiority of adjunctive LDN over placebo for the treatment of MDD. Full details of the study design and methods are described in the published study protocol (Plank et al., 2022a). This study was approved by the Health and Disability Ethics Committee (reference number 2022 FULL 12781) and was prospectively registered in the Australian New Zealand Clinical Trials Registry. This trial was performed in accordance with International Council for Harmonization Good Clinical Practice (ICH GCP); electronic informed consent was obtained by an investigator for all participants before screening.

### 2.2 Participants

Adult participants were recruited from the greater Auckland area through advertisements in general practices, pharmacies, noticeboards, and online social media platforms. A clinician with experience in psychiatry determined eligibility to participate. Participants were required to be at least moderately depressed at the time of screening, defined as a Montgomery-Asberg Depression Rating Scale (MADRS) score greater than or equal to 18 (Montgomery and Asberg, 1979). Participants were also required to be receiving antidepressant medication, but not have greater than Stage II antidepressant resistance as defined by Thase and Rush (Thase and Rush, 1997). A diagnostic interview was used to confirm current MDD and identify exclusionary psychiatric co-morbidities according to the DSM-5 criteria. Medical history was ascertained through a clinical interview to exclude participants with physical comorbidities such as inflammatory diseases, autoimmune diseases, or infections. A minor amendment was made to the wording of our exclusion criteria after recruiting 9 participants (LDN = 5, Placebo = 4) to broaden investigator discretion in excluding only inflammatory illnesses likely to confound immunological measures.

Prior to randomization, hsCRP was measured up to three times, at least 1 week apart, using an external accredited laboratory. Participants with two consistent CRP measures ≤1 mg/L or ≥3 mg/L were included in the study and were subsequently stratified into a low-inflammatory depression and high-inflammatory depression stratum, respectively.

### 2.3 Intervention

LDN (1.5 mg) and placebo capsules appeared identical and were supplied by CompoundLabs Pharmacy Ltd., Auckland. Participants were instructed to take 1 capsule orally once daily at night for 1 week, then 2 capsules at night for 1 week, then 3 capsules (equivalent to 4.5 mg LDN) thereafter. Participants were re-titrated on commencement of the open-label phase. Doses were adjusted in the event of side effects as per the study protocol (Plank et al., 2022a).

### 2.4 Outcomes and questionnaires

MADRS was assessed at baseline and 2, 4, 8, and 12 weeks after commencing treatment. The primary efficacy measure was the change in MADRS score at 12 weeks in LDN compared to placebo, as per the study protocol (Plank et al., 2022a). To avoid unblinding, MADRS raters had no other contact with trial participants.

Blood samples were collected at baseline and 12 weeks for analysis of hsCRP. Samples were collected in a 10 mL EDTA tube. The 10 mL sample was centrifuged at 4 °C and 1.5 RCF for 15 min. Plasma was pipetted from the sample into 8 × 150 μL and 2 × 300 μL aliquots and stored at −80 °C. Plasma hsCRP was analyzed from aliquots by immunoturbidimetry using a Cardiac CRP (Latex) High Sensitive (CRPHS) kit (Roche Diagnostics GmbH, Mannheim; *n* = 36) and a Tina-quant Cardiac hsCRP III kit (Roche Diagnostics GmbH, Mannheim; *n* = 1) in the Roche C311 automatic analyzer.

The Beck Depression Inventory (BDI-II; (Beck et al., 1996)) and Behavioral Activation for Depression Scale (BADS; (Kanter et al., 2007)) were assessed at baseline, 2, 4, 8 and 12 weeks. The Profile of Mood States (POMS; (McNair et al., 1971)), the SF-36 health survey (Ware and Sherbourne, 1992), and the Sickness Questionnaire (SicknessQ; (Andreasson et al., 2018)) were assessed at baseline and 12 weeks. These exploratory outcome measures were collected to enable wider assessment of the effects of LDN on self-reported depression, behavioral activation, mood states, quality of life and sickness-like symptoms in MDD.

The General Assessment of Side Effects (GASE) was completed at 1, 2, 4, 8 and 12 weeks after commencing treatment (Rief et al., 2011). The Stanford Expectation of Treatment Scale (SETS) was also used to assess expectancy effects at baseline (Younger et al., 2012).

### 2.5 Sample size

Given the limited previous studies testing LDN as an intervention for MDD, the sample size was determined based on pragmatic considerations, such as cost and recruitment capacity. Sensitivity analysis of the primary outcome for a fixed sample of 48 (24 participants per treatment group; 4 dropouts with Missing at Random [MAR] data, *α* = 0.05, [1−β] = 0.8) is outlined in the published protocol (Plank et al., 2022a). In brief, this sample size demonstrated sensitivity to detect a change of 6 MADRS points between groups, comparing favorably with the previous study of LDN in MDD, which showed a 10-point difference in change between groups (Mischoulon et al., 2017).

### 2.6 Randomization and blinding

Following hsCRP stratification, participants were randomized 1:1 within each stratum to receive LDN or inactive placebo (microcrystalline cellulose) for 12 weeks, followed by 12 weeks of open-label LDN. Computer-generated randomization was performed by an unblinded investigator, with an initial block size of 12, which was revised to 6 after the enrollment of 25 participants. Participants and study personnel, bar the randomizer and independent pharmacist, were unaware of the allocation to maintain double-blind conditions. Participants were also blinded to their inflammatory stratum. Unblinding of personnel to the study intervention occurred after the final participant in each block completed the primary endpoint at 12 weeks. All missing primary outcome data were classified as MAR or Missing Not at Random (MNAR) for each participant prior to deblinding.

### 2.7 Statistical analysis

Full details of the statistical analysis of the NALDEP trial are included in the Statistical Analysis Plan, which was completed prior to deblinding (Supplementary Material). All statistical analyses were conducted using R statistical software (v4.1.2; R Core Team 2021). Statistical significance was set at *p* < 0.05. hsCRP data were log-transformed for analysis. A small constant of 0.025 was added to the data prior to log transformation to handle zero values.

The effect of LDN versus placebo on the primary outcome (MADRS score), secondary outcome (hsCRP levels), and exploratory outcomes (BDI-II, BADS, SF-36, SicknessQ and POMS) at 12 weeks in the modified intention-to-treat (mITT) population were assessed using linear mixed models adjusted for baseline (Twisk et al., 2018). The mITT population included all participants who received any study drug; mixed-effects modelling handled missing outcome data under a MAR assumption. To improve estimation accuracy, data from 2, 4, and 8 weeks were also included in the model, where available; however, their interpretation was considered exploratory and in need of replication. A secondary analysis of the primary outcome (MADRS score) was conducted in the per-protocol population. This population included all participants who adhered to the protocol criteria and had both baseline and 12-week data. A safety analysis was conducted using GASE data in the safety population, which was identical to the mITT population in terms of sample size for analysis; data were summarized using descriptive statistics.

Pre-specified subgroup analyses (change in MADRS score at 12 weeks in the high-inflammatory group versus the low-inflammatory group) were conducted using two linear mixed models. The first of these models assessed the three-way interaction of time, group and log(baseline hsCRP) but was likely underpowered. The second model included time, group, log(baseline hsCRP), and group-by-log(baseline hsCRP) interaction; this model was better powered but assumed no group-by-time interaction effect (Baune et al., 2021). For all analyses, baseline log-transformed hsCRP was used rather than the pre-randomization inflammatory strata to improve statistical power.

## 3 Results

### 3.1 Participants

**FIGURE 1:**
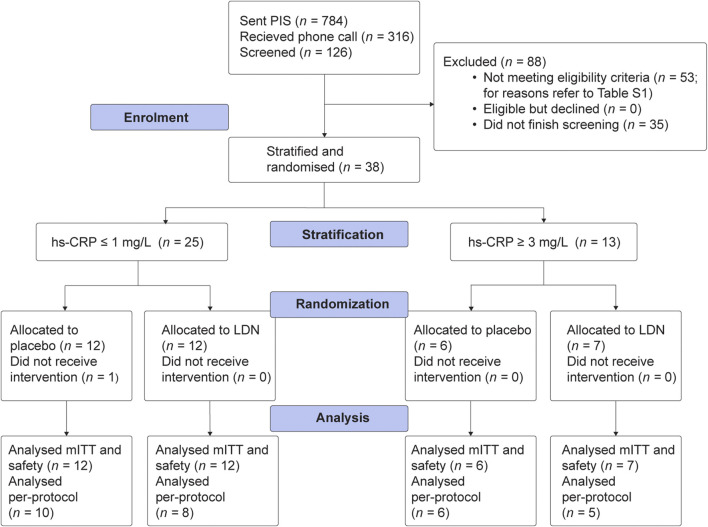
CONSORT diagram outlining participant flow throughout the trial.

Recruitment and data collection occurred between September 2022 and July 2025 at the University of Auckland, New Zealand. A total of 38 participants were enrolled and randomized into the study, and 37 commenced dosing, as shown in the study CONSORT diagram (Figure 1). Of 126 participants who gave informed consent for screening, 53 were ineligible for reasons outlined in Supplementary Table S1. The trial was concluded before the planned sample size was reached due to slow recruitment into the high-inflammatory stratum. Baseline characteristics of the mITT population are outlined in Table 1. All participants were compliant with the intervention (Supplementary Table S2).

**TABLE 1:** Demographic and baseline characteristics of participants in the mITT population.

Characteristic	Summary statistics	Placebo (*n* = 18)	LDN (*n* = 19)
Female	*n* (%)	12 (67)	14 (74)
Age (years)	Median (IQR)	28.5 (25.3, 31.0)	28.0 (23.5, 35.0)
Alcohol (units/week)	Median (IQR)	1 (0, 2)	2 (0, 4)
BMI (kg/m2)	Median (IQR)	26.0 (22.5, 29.2)	26.0 (22.8, 32.5)
Baseline hsCRP (mg/L)	Median (IQR)	0.50 (0.28, 4.94)	0.80 (0.57, 3.35)
*Ethnicity*	n (%)	​	​
NZ European	​	13 (72.2)	15 (78.9)
Other^a^	​	5 (27.8)	4 (21.1)
*Highest level of education*	n (%)	​	​
PhD or masters	​	5 (27.8)	2 (10.5)
Bachelor’s or some tertiary	​	8 (44.4)	12 (63.2)
High school or below	​	5 (27.8)	5 (26.3)
Baseline MADRS	Mean (SD)	23.1 (5.6)	23.3 (5.3)
	Range (min-max)	14–37	15–35
Illness duration (years since onset)^b^	Mean (SD)	13.3 (7.6)	10.9 (6.5)
	Range (min-max)	1–24	2–27
*Current antidepressant*	n (%)	​	​
SSRI	​	12 (66.7)	13 (68.4)
SNRI	​	6 (33.3)	6 (31.5)
Other^c^	​	0	2 (10.5)
*Co-morbidities* ^d^	n (%)	​	​
Anxiety disorder	​	11 (60.1)	10 (52.6)

^a^ Other ethnicities included Māori (*n* = 1), Asian (*n* = 5), and Middle Eastern, Latin American, and African (*n* = 3).

^b^ Illness duration data were unavailable for 1 participant in the placebo group and 1 participant in the LDN group.

^c^ Other antidepressant regimens included mirtazapine (*n* = 1), and mirtazapine + SNRI (*n* = 1).

^d^ *n* = 3 also had post-traumatic stress disorder.

IQR, interquartile range; BMI, body mass index; MADRS, Montgomery Asberg Depression Rating Scale; SD, standard deviation; SSRI, selective serotonin reuptake inhibitor; SNRI, serotonin-norepinephrine reuptake inhibitor.

Treatment was discontinued by 6 participants in the LDN group and 2 participants in the placebo group. Treatment discontinuation in the LDN group occurred between 0 and 2 weeks (*n* = 3), 4 and 8 weeks (*n* = 1) and 8 and 12 weeks (*n* = 2); and, in the placebo group occurred between 2 and 4 weeks. Study-related reasons for treatment discontinuation in the LDN group (*n* = 3; 15.8%) included headache (*n* = 2) and initiation of exclusionary medicine during the study (*n* = 1), and in the placebo group (*n* = 2; 11.1%) rash (*n* = 1) and panic attacks (*n* = 1).

No serious adverse events occurred during the trial. Adverse events self-reported as related to the intervention are presented by group in Table 2; data for all reported adverse events are available in Supplementary Tables S3 and S4. The most frequent events experienced in the LDN group included nightmares/abnormal dreams (*n* = 8, 42.1%), sleep problems (*n* = 7, 36.8%), and headaches (*n* = 5, 26.3%). Adjustments to the dosing and titration schedule to mitigate adverse events were accessed by 6 participants in the LDN group and 3 participants in the placebo group. This included slower up-titration (LDN = 3, placebo = 2) and changing from nighttime dosing to morning dosing (LDN = 3, placebo = 1).

**TABLE 2:** Adverse events self-reported as intervention-related during the double-blind placebo-controlled phase.

Event	Placebo *n participants*	LDN *n participants*	Event severity					
			Mild *n participants*		Moderate *n participants*		Severe *n participants*	
			PLA	LDN	PLA	LDN	PLA	LDN
Gastrointestinal disorders								
Dry mouth	4	4	2	3	1	1	1	0
Increased appetite	3	2	2	2	1	0	0	0
Nausea	0	4	0	3	0	1	0	0
Decreased appetite	4	2	3	1	1	1	0	0
Musculoskeletal and connective tissue disorders								
Muscle pain	2	3	2	1	0	2	0	0
Nervous system disorders								
Dizziness	2	3	2	1	0	2	0	0
Headache	5	5	5	1	0	2	0	2
Fatigue	6	2	3	1	3	1	0	0
Psychiatric disorders								
Agitation	5	1	3	1	2	0	0	0
Anxiety, fearfulness	5	2	1	2	4	0	0	0
Depressed mood	4	3	0	1	4	2	0	0
Nightmare, or abnormal dreams	3	8	2	6	1	1	0	1
Irritability, nervousness	4	2	1	1	3	1	0	0
Insomnia, sleeping problems	5	7	2	6	3	1	0	0

Adverse events presented here were recorded via the GASE questionnaire at weeks 1, 2, 4, 8, and 12. Severity reflects the highest rating reported between baseline and week 12. Events reported by ≥ 15% of participants in either group are presented.

LDN, low-dose naltrexone; PLA, placebo.

Participants were successfully blinded throughout the study; 45% of participants correctly guessed their allocation, indicating that they were no better than chance at guessing whether they were on LDN or placebo. There were also no differences between treatment groups in terms of positive or negative expectations of treatment as measured by the SETS questionnaire at baseline. Blinding and expectancy data are available in Supplementary Table S5.

### 3.2 Primary outcome

**FIGURE 2:**
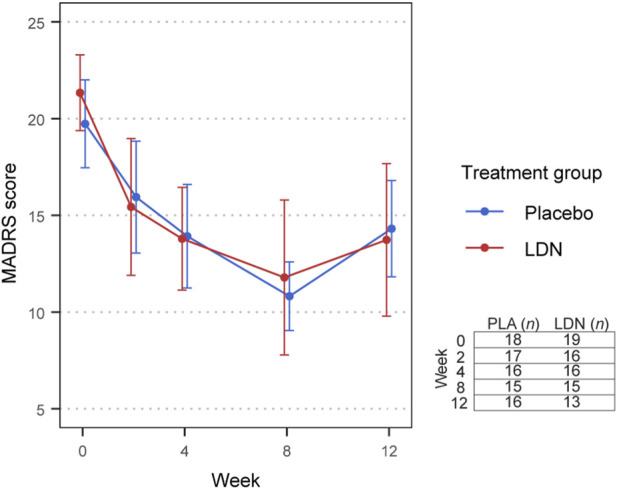
Change in MADRS score over time in response to LDN versus placebo in the mITT population. Plotted values are the mean and error bars are the 95% confidence interval. The non-missing sample size at each time point is tabulated in the bottom right of the figure. Data were analyzed using a linear mixed effects model adjusted for baseline.

At 12 weeks, MADRS scores (M ± SD) were reduced by 10.5 ± 5.6 in the LDN group and 9.8 ± 5.9 placebo group compared to baseline (Figure 2). The mean difference (LDN-placebo) in MADRS score at 12 weeks, adjusted for baseline, was −0.096 (95% CI: −4.420, 4.228), which was not statistically significant (*p* = 0.965), indicating no superior effect of LDN over placebo (Table 3). The changes in MADRS score between treatment groups at 2, 4 and 8 weeks compared to baseline were also not statistically significant. Analysis in the per-protocol population was broadly consistent with the mITT findings, although a statistically significant difference (p = 0.036) in MADRS score at 8 weeks compared to baseline, favoring placebo, was observed. Age, sex, and log(baseline hsCRP) were evaluated as potential covariates; however, none demonstrated a confounding effect on MADRS scores (Supplementary Table S6).

**TABLE 3:** Mean differences (LDN-placebo) in the change in MADRS scores between treatment groups at each time point.

Timepoint	mITT			Per-protocol		
	Adjusted mean difference	95% CI	*p* value	Adjusted mean difference	95% CI	*p* value
2 weeks	−1.616	−5.678, 2.446	0.437	−2.650	−7.053, 1.754	0.240
4 weeks	1.397	−2.726, 5.520	0.508	1.779	−2.570, 6.128	0.424
8 weeks	3.813	−0.427, 8.054	0.080	4.750	0.346, 9.153	0.036
12 weeks	−0.096	−4.420, 4.228	0.965	−0.053	−4.402, 4.296	0.981

Linear mixed models were adjusted for baseline and included timepoint dummy variables and interaction terms between timepoint and treatment allocation as fixed effects, and participants as a random effect. The primary outcome measure was the difference between the LDN and placebo groups at 12 weeks in the mITT population. All model assumptions were checked using Q-Q and residuals versus fitted values plots.

mITT, modified intention-to-treat; CI, confidence interval.

### 3.3 Secondary outcomes and exploratory outcomes

There was no evidence for an effect of LDN on log(hsCRP) level or any exploratory questionnaires BDI-II, BADS, SF-36, POMS and SicknessQ - at 12 weeks (Supplementary Table S7).

### 3.4 Subgroup analyses

**FIGURE 3:**
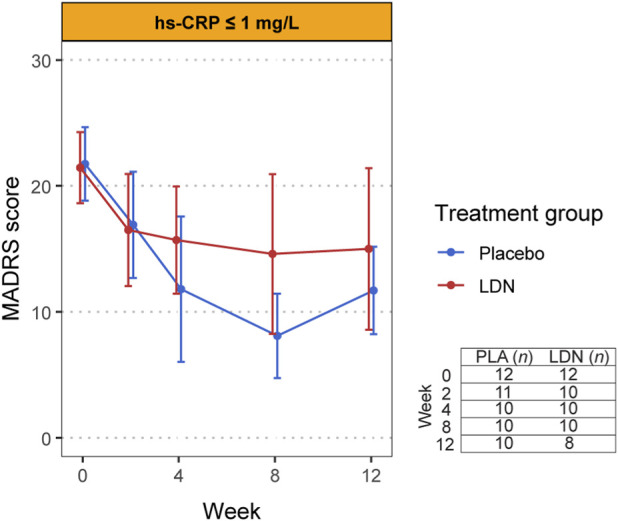
MADRS scores over time in response to LDN versus placebo in the low hsCRP strata Plotted points are the mean, and error bars are the 95% confidence interval. The non-missing sample size at each time point is tabulated in the bottom right of the figure.

**FIGURE 4:**
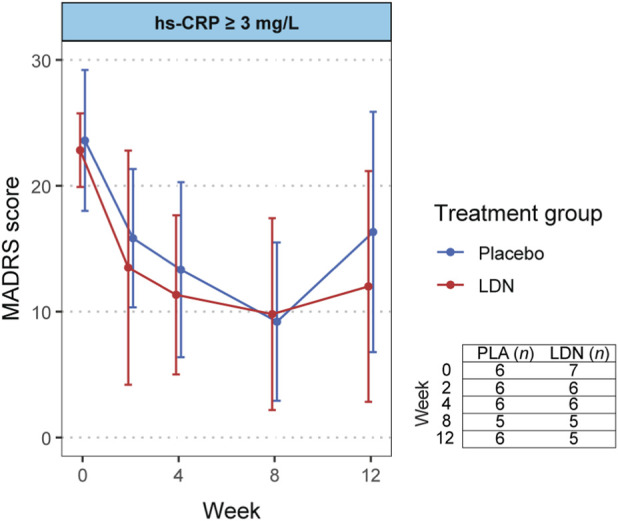
MADRS scores over time in response to LDN versus placebo in the high hsCRP strata. Plotted points are the mean, and error bars are the 95% confidence interval. The non-missing sample size at each time point is tabulated in the bottom right of the figure.

Subgroup analyses showed no evidence of a moderating effect of log(baseline hsCRP) on change in MADRS score. In the first model, the 3-way group-by-time-by-log(baseline hsCRP) interaction effect was not statistically significant (*p*_*interaction*_ = 0.633), indicating no evidence that the effect of the intervention at 12 weeks differed by baseline hsCRP. In the second model, which assumed no group-by-time interaction effect, the group-by-log(baseline hsCRP) interaction effect was also not statistically significant (*p*_*interaction*_ = 0.625), indicating that, across the study, the difference between groups did not vary depending on baseline hsCRP. As illustrated in Figures 3, 4, the hsCRP stratified parallel arms of the study showed a comparable reduction in MADRS score over time.

## 4 Discussion

This is the second clinical trial assessing the antidepressant effects of adjunctive LDN in MDD to date. LDN was well-tolerated throughout the study, and participants were well-blinded and compliant with the intervention. We found no evidence that 12 weeks of LDN was superior to placebo in the adjunctive treatment of moderate MDD, in contrast with previous literature (Mischoulon et al., 2017). LDN did not reduce hsCRP levels or alter scores on exploratory questionnaires–BDI-II, BADS, SF-36, SicknessQ, and POMS–indicating a lack of broader effects on systemic inflammation, self-reported depression, behavioral activation, quality of life, sickness behaviors, and mood states.

Several factors may have contributed to the differences observed between the current study and the prior pilot trial. We had a larger sample size (*n* = 37) compared to the pilot trial (*n* = 12). We also trialed a longer treatment period of 12 weeks, compared to 3 weeks, to allow sufficient time to observe the anti-inflammatory benefits of LDN (Parkitny and Younger, 2017; Smith et al., 2011). However, even at the 2 week and 4 week time points, we did not observe a superior effect of LDN on total MADRS score. Some characteristics of our cohort also differed from the pilot trial; participants in our study were younger, had lower MADRS scores at baseline, and were not exclusively treated with pro-dopaminergic antidepressant regimens. Our study also employed additional exclusion criteria to limit treatment resistance and immunological confounds, thereby reducing sample heterogeneity. There was a similar proportion of female participants in our cohort compared to the pilot trial.

A growing body of evidence suggests that inflammation and dopaminergic signaling are mechanistically linked in MDD. For example, positron-emission tomography (PET) evidence has shown that IFN-α administration is associated with diminished dopamine availability in the striatum, concomitant with reduced motivation (Capuron et al., 2012) and ameliorable with levodopa administration (Felger et al., 2015). The present study excluded participants taking dopaminergic agonists and stimulants to differentiate the anti-inflammatory effects of LDN in MDD from its dopamine-sensitizing effects, which were the subject of the prior pilot trial. This approach may have limited the antidepressant effects of LDN; an *in vitro* study has shown that structurally related opioid antagonist naloxone protects dopaminergic neurons by inhibiting the neuroinflammatory response (Bekhbat et al., 2025; Bekhbat et al., 2022). Due to our small sample size, we could not conduct subgroup analyses on antidepressant type to determine if those on dopaminergic antidepressants responded preferentially to LDN. Future trials that are adequately powered to look at the differential effects of LDN adjunctive to dopaminergic versus non-dopaminergic antidepressants are needed.

LDN appears to exert anti-inflammatory effects in the CNS via antagonism at toll-like receptors (Cant et al., 2017; Hutchinson et al., 2008) and modulation of the opioid growth factor axis (Donahue et al., 2011; Hammer et al., 2016), resulting in reduced glial activation, cytokine and neurotoxic superoxide release, and neuroimmune cell proliferation (Grace et al., 2015; Kucic et al., 2021; Liu et al., 2000a; Liu et al., 2000b). Past studies suggest that LDN reduces symptoms in conditions with neuroinflammation, such as fibromyalgia (Younger et al., 2013; Younger and Mackey, 2009; Bruun-Plesner et al., 2020) and multiple sclerosis (Cree et al., 2010). However, we did not observe any reduction in symptoms in MDD. Past evidence associates neuroinflammation with unmedicated (Setiawan et al., 2015), treatment-resistant (Attwells et al., 2020), severe (Setiawan et al., 2015), and suicidal MDD (Martinez et al., 2012; Holmes et al., 2018). As the current study limited these factors, neuroinflammation in our cohort may not have been substantial enough to benefit from LDN’s effects. Future studies should examine LDN in severe, treatment-resistant or unmedicated MDD, or in MDD comorbid with inflammatory conditions, where its effects may be more impactful.

Our study used stratified randomization to recruit individuals with MDD and chronic low-grade inflammation, as evidence suggests these patients are more likely to benefit from anti-inflammatory therapy (Arteaga-Henriquez et al., 2019). We used hsCRP as the marker of inflammation; however, we did not find that it moderated treatment response. A post-hoc observation of our data shows the high-inflammation stratum had a higher average BMI compared to the low-inflammation stratum (data not shown). This is relevant because BMI and related metabolic pathways, such as insulin resistance, atherosclerosis, metabolic syndrome and platelet dysfunction (Timpson et al., 2011; Blaha et al., 2011), are non-neural sources of inflammation that may not be amenable to LDN. Therefore, while hsCRP may be an appropriate stratification tool for trials targeting systemic or immunometabolic depression pathways (Zwiep et al., 2025), it is unlikely to have sufficient sensitivity or specificity to be used in trials targeting neuroinflammatory pathways.

There remains a lack of validated tools to assess treatment effects for neuroinflammation in MDD. PET can detect neuroimmune cell activation *in vivo*; however, it is costly and involves exposure to ionizing radiation (Gritti et al., 2021). Cytokine sampling (e.g., IL-6, TNF-α and IL-1) has been performed frequently in MDD studies (Miller et al., 2025); however, serum sampling poorly reflects central effects and cerebrospinal fluid sampling is highly invasive. Moreover, accurate quantification of cytokines is challenging owing to a lack of standardized assay methods to reliably detect the very low concentrations present in psychiatric disorders (Miller et al., 2025). Future research is ultimately needed to identify and validate noninvasive markers of neuroinflammation. Novel magnetic resonance imaging markers such as brain temperature derived from echo-planar spectroscopic imaging and white matter microstructure derived from advanced diffusion-weighted imaging are emerging as promising candidates for this (Plank et al., 2022b; Plank et al., 2025).

A strength of the present study is that it is the largest RCT conducted to date evaluating the efficacy of adjunctive low-dose naltrexone for MDD and incorporated hsCRP sampling to assess potential mechanisms of effects. We did not reach our target sample size of 48 (24 per treatment group); post-hoc analysis showed the final dataset (n = 37, including 8 dropouts) was powered to detect a larger mean difference of approximately 8 MADRS points. Although the present study is limited in its ability to detect small but clinically significant differences, it remains better powered to detect an effect compared to the pilot trial (Mischoulon et al., 2017).

Attrition of participants was a limitation; treatment was discontinued by 8 participants (22%; LDN = 6, placebo = 2), which included 6 participants (13%; LDN = 3, placebo = 2) judged to be missing not at random (MNAR). As less than 15% of the data were NMAR, we did not employ imputation techniques to investigate the sensitivity of the primary outcome to missing data. However, given that groups were relatively balanced in terms of the proportion of MNAR data and the reasons for missingness were varied and not clearly associated with treatment group or depression severity, we judge the likelihood of bias in the effect estimate due to the characteristics of people with MNAR data to be low.

In conclusion, this study did not find evidence that LDN is an effective adjunctive treatment for moderate MDD and calls into question the utility of hsCRP as a stratification marker in trials of centrally acting anti-inflammatory interventions in MDD. Larger RCTs of LDN in MDD are needed, and these should be conducted in patients with more severe, treatment-resistant depression or in MDD comorbid with inflammatory illness. These studies should utilize novel stratification markers and outcome measures that are more sensitive and specific to neuroinflammatory pathways than hsCRP.

## Data Availability

The raw data supporting the conclusions of this article will be made available by the authors, without undue reservation.
