# Supplementary material for: Low-dose naltrexone as an adjunctive treatment for major depressive disorder: findings from a randomized, double-blind, placebo-controlled hybrid parallel-arm study
Source: Front Pharmacol. 2026 Mar 6;17:1767654. doi: 10.3389/fphar.2026.1767654 (PMC13002618; doi:10.3389/fphar.2026.1767654)
Supplement: Supplementary file 1 [file Supplementaryfile1.zip › Statistical Analysis Plan.DOCX]

Statistical Analysis Plan

Low-dose NALtrexone as an adjunctive treatment in major DEPressive disorder (NALDEP)

| SAP VERSION | 1.1 |
| --- | --- |
| SAP VERSION DATE | 3^rd^ September 2024 |
| TRIAL STATISTICIAN | Dr Alana Cavadino |
| Protocol Version (SAP associated with) | Protocol Version 4.4.1 |
| TRIAL PRINCIPAL INVESTIGATOR | Dr Joanne Lin |
| SAP AUTHOR(s) | Ben Moloney, AP Suresh Muthukumaraswamy, Dr Alana Cavadino |

#

# SAP Signatures

I give my approval for the attached SAP entitled “Low-dose NALtrexone as an adjunctive treatment in major DEPressive disorder (NALDEP)” dated 3^rd^ September(Version 1.1)

**Statistician (Author)**

Name: Dr Alana Cavadino

Signature:    4^th^ September 2024                         

Date:      
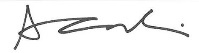


**Principal Investigator**

Name: Dr Joanne Lin

Signature:            
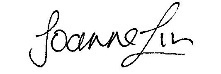
                                    

Date:         25^th^ September 2024

# Table of Contents

[1 SAP Signatures 2](#_Toc176265317)

[2 Table of Contents 3](#_Toc176265318)

[3 Abbreviations and Definitions 5](#_Toc176265319)

[4 Introduction 6](#_Toc176265320)

[4.1 Preface 6](#_Toc176265321)

[4.2 Scope of the analyses 6](#_Toc176265322)

[5 Study Objectives and Endpoints 7](#_Toc176265323)

[6 Study Methods 7](#_Toc176265324)

[6.1 General Study Design and Plan 7](#_Toc176265325)

[6.2 Inclusion-Exclusion Criteria and General Study Population 8](#_Toc176265326)

[6.2.1 Inclusion Criteria 8](#_Toc176265327)

[6.2.2 Exclusion Criteria 8](#_Toc176265328)

[6.2.3 Lifestyle Considerations 9](#_Toc176265329)

[6.3 Randomisation and Blinding 9](#_Toc176265330)

[6.4 Study Assessments 9](#_Toc176265331)

[6.4.1 Description of Efficacy Endpoints 10](#_Toc176265332)

[6.4.2 Timing of Efficacy Endpoints 10](#_Toc176265333)

[6.4.3 Description of Safety Endpoints 10](#_Toc176265334)

[6.4.4 Timing of Safety Endpoints 11](#_Toc176265335)

[7 Sample Size 11](#_Toc176265336)

[8 General Analysis Considerations 11](#_Toc176265337)

[8.1 Timing of Analyses 11](#_Toc176265338)

[8.2 Analysis Populations 11](#_Toc176265339)

[8.2.1 Modified Intention to Treat (Full Analysis Set) 11](#_Toc176265340)

[8.2.2 Per Protocol Population 12](#_Toc176265341)

[8.2.3 Safety Population 12](#_Toc176265342)

[8.3 Covariates and Subgroups 12](#_Toc176265343)

[8.4 Missing Data 12](#_Toc176265344)

[8.5 Interim Analyses and Data Monitoring 13](#_Toc176265345)

[8.6 Multiple Testing 13](#_Toc176265346)

[9 Summary of Study Data 13](#_Toc176265347)

[9.1 Subject Disposition 13](#_Toc176265348)

[9.2 Derived variables 14](#_Toc176265349)

[9.3 Protocol Deviations 15](#_Toc176265350)

[9.4 Demographic and Baseline Variables 15](#_Toc176265351)

[9.5 Concurrent Illnesses and Medical Conditions 16](#_Toc176265352)

[9.6 Treatment Compliance 16](#_Toc176265353)

[10 Efficacy Analyses 16](#_Toc176265354)

[10.1 Primary Efficacy Analysis 16](#_Toc176265355)

[11 Safety Analyses 17](#_Toc176265356)

[11.1 Adverse Events 17](#_Toc176265357)

[11.2 Deaths, Serious Adverse Events and other Significant Adverse Events 17](#_Toc176265358)

[11.3 Pregnancies 17](#_Toc176265359)

[12 Other Analyses 17](#_Toc176265360)

[13 Reporting Conventions 17](#_Toc176265361)

[14 Quality Assurance of Statistical Programming 18](#_Toc176265362)

[15 Summary of Changes to the Protocol and/or SAP 18](#_Toc176265363)

[16 References 19](#_Toc176265364)

[17 Listing of Tables, Listings and Figures 19](#_Toc176265365)

#

# Abbreviations and Definitions

| AE | Adverse Event |
| --- | --- |
| BMI | Body Mass Index |
| CRP | C-reactive protein |
| GASE | General Assessment of Side Effects |
| LDN | Low-dose naltrexone |
| MADRS | Montgomery-Asberg Depression Rating Scale |
| MDD | Major Depressive Disorder |
| mITT | Modified Intention to Treat |
| PIS | Participant Information Sheet |
| SAE | Serious Adverse Event |
| SAP | Statistical Analysis Plan |

#

# Introduction

## Preface

As many as 280 million individuals are estimated to suffer from depression worldwide; and depression is a major contributor to the overall global burden of disease (World Health Organisation). Major Depressive Disorder (MDD) is characterised by a persistently low mood, or lack of enjoyment in previously enjoyable activities, accompanied by symptoms such as lack of energy, problems sleeping, an observable slowing of thoughts and movements, change in appetite, problems concentrating, feelings of worthlessness, excessive guilt, and suicidal ideation (American Psychiatric Association, 2013). All of which can severely impact an individual’s ability to function and contribute to a reduction in quality of life. Current treatments for MDD are ineffective in approximately one-third of patients resulting in a large economic burden and reduced quality of life for a significant proportion of the global population (The Royal Australian and New Zealand College of Psychiatrists, 2016).

Current pharmacological therapies for MDD primarily target the monoaminergic systems; based on the theory that depression is due to a deficiency in monoaminergic neurotransmission (Hindmarch, 2001). Conventional treatments such as selective-serotonin-uptake-inhibitors (SSRIs), monoamine oxidase inhibitors (MAOIs), tricyclic antidepressants, and selective noradrenaline reuptake inhibitors (SNRIs), all target the monoaminergic system in various mechanisms. Recent research demonstrates, however, that depression is influenced by factors beyond monoamines, such as neuroinflammation (Bullmore, 2018; Raison et al., 2006). Investigating novel anti-inflammatories is of particular importance due to high levels of high sensitivity C-reactive protein (hs-CRP), a peripheral indicator of inflammation, being associated with treatment resistant depression (Chamberlain et al., 2019).

The current study aims to explore the potential role of low-dose naltrexone (LDN), a drug with purported anti-inflammatory properties in the central nervous system, as an adjunctive treatment in patients with MDD. Moreover, blood samples and MRI techniques, including magnetisation transfer imaging, diffusion-weighted imaging and magnetic resonance spectroscopy, may help to elucidate the inflammatory mechanisms of action in MDD, and the neurobiological mechanism of LDN. Clinical assessments, questionnaires, a cognitive test battery and electroencephalography will also be performed to explore potential biomarkers of inflammatory depression.

## Scope of the analyses

The analyses described in this SAP will assess the efficacy and safety of low-dose naltrexone in comparison with placebo (microcrystalline cellulose) and will be included in the clinical study report, student theses and academic publications. The analyses described cover only those for the primary and safety endpoints described in the Protocol. Analyses of secondary and tertiary/exploratory objectives are not included and will be analyzed subsequently, and in the case of tertiary/exploratory objectives will be considered exploratory/hypotheses generating analyses.

# Study Objectives and Endpoints

The primary objective of the NALDEP study is to test whether LDN can modify depressive symptomatology in patients with MDD relative to placebo. Primary, secondary and safety objectives/endpoints are detailed in the NALDEP protocol section 3.

# Study Methods

## General Study Design and Plan

This Phase 4 trial is a randomised, double-blind, placebo-controlled, hybrid parallel arm study investigating the potential of LDN as an adjunctive treatment for MDD. Participants who meet the inclusion criteria following an initial screening will be prospectively stratified into low/high inflammatory status (n=24 per group) based on levels of hs-CRP. There are four study arms, two for the high inflammation group (n=24), and two for the low inflammation group (n=24). One arm from each group will receive LDN for the first 12 weeks (n=12), and the other will receive placebo for the first 12 weeks (n=12), as shown in the figure below. After the initial 12 weeks, there will be a further 12 weeks where all participants will receive LDN allowing us to check the durability and efficacy of LDN as a treatment for MDD.

Figure 1: Flowchart of participant stratification based on level of high sensitivity C-reactive protein (hsCRP).

**Participants with MDD** receiving antidepressant therapy.

**Healthy controls**

**Low inflammatory state**

hsCRP $\leq$ 1mg/L (*n = 24)*

**High inflammatory state**

hsCRP $\geq$ 3mg/L (*n = 24)*

**LDN**

*(n =12; 12 weeks)*

**Placebo**

*(n =12; 12 weeks)*

**LDN**

*(n =12; 12 weeks)*

hsCRP $\leq$ 1mg/L

**Timeline:**

**0 weeks**

**24 weeks**

**12 weeks**

**Placebo**

*(n =12; 12 weeks)*

**LDN**

*(n =24; 12 weeks)*

**LDN**

*(n =24; 12 weeks)*

Initial measurement of outcomes

Endpoints of outcome measures

## Inclusion-Exclusion Criteria and General Study Population

### Inclusion Criteria

In order to be eligible to participate in this study, an individual must meet all of the following criteria:

1. Provision of signed and dated informed consent form
2. Stated willingness to comply with all study procedures and availability for the duration of the study
3. Male or female, aged 18 to 55 years.
4. Diagnosis of MDD as per the Diagnostic and Statistical Manual 5 criteria for MDD
5. Experiencing significant depressive symptoms, as indicated by a score ≥18 on the MADRS
6. Receiving treatment with an antidepressant agent (not greater than Stage II antidepressant resistance (Thase & Rush, 1997))
7. Clinically stable for at least four weeks
8. hs-CRP levels either ≥ 3 mg/L or ≤ 1 mg/L
9. Ability to take oral medication and be willing to adhere to the dosing regimen
10. Agreement to adhere to Lifestyle Considerations

### Exclusion Criteria

An individual who meets any of the following criteria will be excluded from participation in this study:

1. Current use of prescription opioid analgesics, psychostimulants, dopamine agonists
2. The current use of any opioid-based drugs recreationally.
3. Any current or past bipolar or psychiatric disorder or psychotic features (other psychiatric conditions such as anxiety will be allowed for individuals with MDD, provided they are not the primary source of the participant’s depressive symptomatology)
4. Acute risk of suicide
5. Any known neurological disorders or neurodegenerative condition
6. Acute infectious pathology, or chronic or acute inflammatory diseases that, in the judgement of the investigators, interferes with study measures or procedures
7. Any clinically significant medical conditions, e.g., renal, hepatic, or cardiovascular conditions, uncontrolled thyroid dysfunction, metabolic disorders, cancer, seizure disorders
8. Uncontrolled autoimmune diseases or severe chronic pain
9. Or any condition that, in the judgement of the investigators, interferes with study measures or procedures
10. Long‐term, frequent anti‐inflammatory or immunosuppressive therapy
11. Contraindications to MRI and blood tests, including those who refuse to be informed of an incidental finding
12. Women who are pregnant or breastfeeding, or women of child-bearing age who are not on a medically acceptable form of contraception
13. Substance use disorders within the previous 12 months
14. Any allergy or intolerance to naltrexone

### Lifestyle Considerations

There will not be any modification to the usual access to care pathways whilst participants are enrolled in the study. We will ask participants to:

- Abstain from alcohol and drugs for 24 hours before the start of each MRI and EEG session.
- Have a plan with their doctor to stay on the same anti-depressant for at least 12 weeks.

## Randomisation and Blinding

Following stratification of the eligible participants into high inflammatory and low inflammatory status, participants will be randomly allocated to the LDN or placebo group. Computer-generated randomisation will be performed by one member of the research team. Blocked randomisation will be used with a block size of 6. Hence there will be eight randomisation blocks in the trial – four for low CRP and four for high CRP.

LDN or placebo will be prescribed by the study psychiatrist and delivered to the participant by the study pharmacist. The randomiser and pharmacist will be the only members of the research team unblinded to the identity of the medication. To maintain double-blind conditions, the participant and study team members conducting the measurements for study outcomes will not be aware of the identity of the medication. At the end of the study during patient debriefing, participants will be asked to identify the medication they think they received. Unblinding of participants to the study intervention will occur only after the final participant, within their randomisation block, has completed the trial. The research team will be unblinded once the final participant in a research block has completed their outcome measurements at the three month mark.

If the participant experiences a reaction or acute deterioration of health during the study and requires medical intervention, or in the event a participant requires opioids for acute analgesia, the identity of the medication may be revealed by an unblinded member of the study team. The randomiser will prepare a set of code break envelopes, which will be made available to an on-call team member 24/7 in the event of an emergency. The participants will be provided with a contact phone number for the study team, and a study team member will be on-call and available 24/7 to break blinding and advise treating health care professionals.

## Study Assessments

A complete list of outcome measures is summarized in the Standard Protocol Items: Recommendations for Interventional Trials (SPIRIT) Figure 2.

###
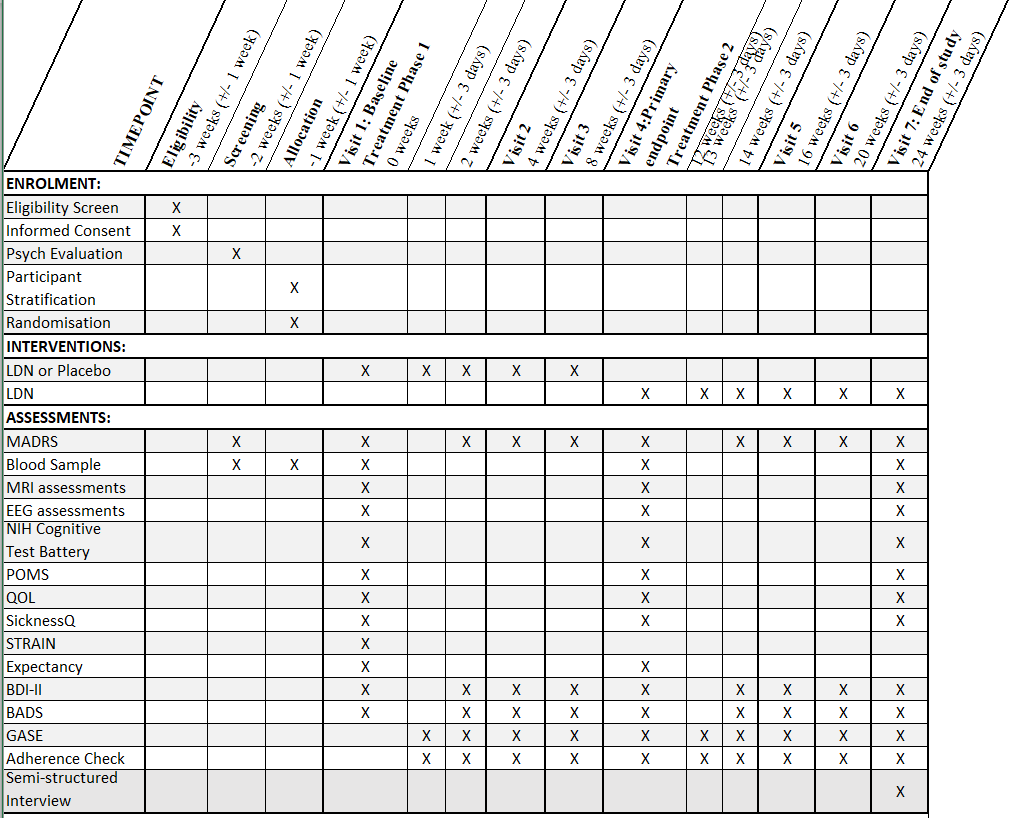
Description of Efficacy Endpoints

Figure 2: Standard Protocol Items: Recommendations for Interventional Trials (SPIRIT)

MADRS

The MADRS is a 10-item clinician-administered outcome that evaluates the core symptoms of depression. Items are rated on a 7-point Likert scale (0=no abnormality to 6=severe). Item responses are summed to give a single score between 0-60, where higher scores indicate greater levels of depression with sum scores treated as a continuous variable. The MADRS is collected at baseline, 2 weeks, 4 weeks, 8 weeks and 12 weeks.

### Timing of Efficacy Endpoints

Efficacy endpoints should be collected within +/- 14 days of the scheduled date. See also Section 9.3 for how deviations will be dealt with.

### Description of Safety Endpoints

General Assessment of Side Effects Questionnaire

The Generic Assessment of Side Effects questionnaire is a structured and validated method for assessing 36 subjective side effects in clinical trials (Rief et al., 2011). For each side effect, participants are prompted to rate severity one a scale of 0 “Not present” to 3 “severe,” and whether they believe it is related to the intervention or not. GASE data is collected at 1 week, 2 weeks, 4 weeks, 8 weeks, and 12 weeks.

### Timing of Safety Endpoints

Safety endpoints should be collected within +/- 14 days of the scheduled date. See also section 9.3 for how deviations will be dealt with.

# Sample Size

Given the relative lack of previous studies using LDN as an intervention for MDD and the desire to explore secondary and exploratory outcomes with maximum power, sample size was based on pragmatic reasons (cost and potential ability to recruit participants). Nevertheless, a sensitivity analysis of the primary outcome was conducted for the fixed sample size of 48 (12 participants per group). Monte Carlo simulations were conducted in R using the mixed effect models described above with 10,000 simulations per run. Data were simulated on each iteration using the following parameters (n= 48, 4 dropouts with data missing at random, α=0.05, (1-β)=0.8, baseline MADRS score of 30, random effect variance = 6.32 and error variance of 4.89 (variances estimates were obtained from linear mixed effect models fit to data from a previous antidepressant trial (Mischoulon et al., 2017)). Monte Carlo simulations revealed that the current study was sensitive to detect changes of ~6 MADRS points. This compares relatively favourably to the previous study of LDN in MDD which showed an 18-point drop in MADRS scores with LDN compared to an 8-point drop under placebo (10-point difference) although this previous study only included 12 participants in total. Overall, the current study is much better powered in terms of primary outcome than the previous pilot study of low-dose naltrexone in MDD although a relatively large effect size is still required for significance to be determined.

# General Analysis Considerations

## Timing of Analyses

The final analysis will be performed when the following criteria are met:

- The last participant has completed their three-month visit in the Schedule of Assessments.
- The final SAP version is agreed and signed.
- The Study database (not including long-term follow-up) is locked.

## Analysis Populations

### Modified Intention to Treat (Full Analysis Set)

All participants who received any study drug. This is a modification to Intention to Treat as participants who are randomised to a group but who dropout prior to receiving any study drug will be replaced (as described in the Study Protocol). Participants who dropout prior to receiving the intervention will retain their Screening identifier but not their Study identifier and will be included in the CONSORT diagram.

### Per Protocol Population

All participants who adhere to the major criteria in the protocol and have data at the Baseline and 12 week time-points. Determination of status will be made on a per-participant basis at the immediately before database lock.

### Safety Population

All subjects who received any study treatment (including control) but excluding participants who drop out prior to receiving any treatment. This population is the same as the mITT population in terms of *n* for analysis.

The primary population for efficacy analyses will be the Modified Intention to Treat population with additional secondary analyses performed on the per protocol population.

The primary population for safety analyses will be the Safety Population with additional analyses performed on the per protocol population.

See Section 9.3 for how major protocol deviations will be managed.

## Covariates and Subgroups

The most likely covariate to have an effect on both primary and secondary efficacy endpoints is Baseline score of the measure. Other potential covariates that might influence these endpoints are sex (categorial), age (continuous) and BMI (continuous). There may also be a differing effect between the CRP-stratified subgroups.

For this reason, Baseline score will be included as a covariate in the Primary Efficacy Analysis described in Section 10.1. Other covariates and analysis within subgroups will be included in Secondary Analyses and are not described in this document.

## Missing Data

The amount of missing data will be quantified/tabulated for each analysis and classified as to whether it was considered Missing at Random or Missing Not at Random (e.g. early discontinuation from the study). It is expected that no data will be missing at Baseline as participants cannot start the intervention without attending their Baseline session, and any missing data should therefore be restricted to post-intervention data.

The mixed effect modelling analysis approach described in Section 10 does not require complete cases and by default missing data is analysed as Missing at Random and this will be the approach used in the primary efficacy analysis. If the degree of Missing Not at Random data is judged to exceed 15% then secondary approaches using imputation techniques will be investigated to test the sensitivity of the primary efficacy analysis to this missing data.

## Interim Analyses and Data Monitoring

An interim efficacy analysis is planned for the purposes of a student thesis. As no adjustment to sample size or trial conduct will be made following the interim analyses, no adjustment to the alpha level will be made.

## Multiple Testing

For the primary endpoint – MADRS score at 12 weeks compared to baseline across the two groups there is a single p-value of interest and no correction for multiple time-points will be made.

# Summary of Study Data

All continuous variables will be summarized from the modified intention to treat set using the following descriptive statistics: n (non-missing sample size), mean, standard deviation, maximum and minimum for each of the two groups (LDN / placebo).

## Subject Disposition

A CONSORT diagram for NALDEP using a format similar to Figure 3. A table listing the reasons for exclusion at Screening will be populated.

Excluded (n= ?)

Not meeting inclusion criteria (n= ?)

Eligible but declined (n= ?)

Didn’t finish screening (n= ?)

Allocated to placebo (n= )

Did not receive intervention (n=)

CRP ≤1mg/L (n=)

CRP ≥3 mg/L (n=)

Allocated to LDN (n= )

Did not receive intervention (n=)

Allocated to placebo (n= )

Did not receive intervention (n=)

Allocated to LDN (n= )

Did not receive intervention (n=)

Analysed mITT and safety (*n* = ?)

Analysed per protocol (n = ?)

Analysed mITT and safety (*n* = ?)

Analysed per protocol (n = ?)

Analysed mITT and safety (*n* = ?)

Analysed per protocol (n = ?)

Analysed mITT and safety (*n* = ?)

Analysed per protocol (n = ?)

Sent PIS (n = ?)

Received phone call (n = ?)

Screened (n= ?)

Stratified and Randomised

**Stratification**

**Analysis**

**Randomisation**

**Enrolment**

Figure 3: Blank CONSORT diagram for NALDEP

## Derived variables

The efficacy variable will be computed as per Section 6.4.1 by simple addition.

## Protocol Deviations

As defined in the study protocol participants are required to take 75% of doses in order to be deemed compliant with the study protocol. Participants who take >=75% of doses can be entered into both the Modified Intention to Treat and Per Protocol Analysis sets. Participants who take <75% of doses can only be entered into the Modified Intention to Treat Analysis Set.

As per Section 6.4.2 efficacy endpoints should be collected within +/- 14 days of the scheduled date. This is required for those datapoints to be entered into the Modified Intention to Treat and Per Protocol Analysis sets. For the 12-week data point, efficacy data collected 14-21 days outside the 12-week collection point will be included into the Modified Intention to Treat dataset but not the Per Protocol Analysis set. Data outside of the 21 day window will be treated as missing.

If two efficacy endpoints are collected within the allowed time-frame the datapoint closest in time will be used for the Analysis and the other data discarded.

## Demographic and Baseline Variables

A demographic table containing at least the data in Table 1 will be constructed.

Table 1: Minimum demographic table from NALDEP

| **Observation** |  | | **LDN** | | **PLA** | |
| --- | --- | --- | --- | --- | --- | --- |
| Age, *M* (*sd*) |  | |  | |  | |
| Sex | Male, n (%) | |  | |  | |
|  | Female, n (%) | |  | |  | |
| Duration of Illness (years since onset) , M(sd) | |  | |  | |  |
| Current Antidepressant,  SSRI *n* (%) | |  | |  | |  |
| SNRI *n* (%) | |  | |  | |  |
| Other *n* (%) | |  | |  | |  |
| Ethnicity |  | |  | |  | |
|  | New Zealand European | |  | |  | |
|  | Māori | |  | |  | |
|  | Pacific Peoples | |  | |  | |
|  | European | |  | |  | |
|  | Asian | |  | |  | |
|  | Middle Eastern/Latin American/African | |  | |  | |

## Concurrent Illnesses and Medical Conditions

Psychiatric co-morbidities will be quantified using the Mini-International Neuropsychiatric Interview conducted at Screening and added to Table 1: Minimum demographic table from NALDEP.

## Treatment Compliance

Participants were asked to return their capsule bottles at the end of each 4 week period and intervention compliance was determined based on returned capsule count. If a participant was unable to attend an in-person visit, the bottle was set aside and returned at the next scheduled visit, or the participant instructed to count and report the remaining capsules in each bottle. The count of remaining capsules for each 4 week period were aggregated over the 12-week duration. Where returned bottle count was not available, self-reported missed doses at the “adherence check” (1, 2, 4, 8 and 12 weeks) were used to determine compliance. Treatment compliance will be summarised as per Table 2.

Table 2 Treatment compliance summary table for NALDEP

| Doses taken (%) | Placebo  N (%) | LDN  N (%) |
| --- | --- | --- |
| >=75% (based on returned capsule count) |  |  |
| <75% (based on returned capsule count) |  |  |
| >=75% (based on self-reported data) |  |  |
| <75% (based on self-reported data) |  |  |

# Efficacy Analyses

## Primary Efficacy Analysis

Analyses of MADRS scores at the 12 week timepoint will be conducted using linear mixed effect models using the modified intention to treat analysis set. The outcome measures MADRS score is a continuous variable while factors of group (LDN / Placebo) and Event (Baseline / 2, 4, 8, 12 weeks) are categorical variables. For modelling, Group and Event will be treated as fixed effects, and Participants as a random effect, with the primary estimate of interest being the Group x Event interaction effect at 12 weeks.

As described by Twisk et al. (2018) in order to ­­properly account for baseline effects (such as imbalance between groups and regression to the mean) in a repeated measures analysis, the treatment variable should not be included in the model but with Event/Group interaction effects included. Although the 12-week measure is the primary comparison of interest, to improve estimation accuracy data from the 2,4,8 measurement time-points will be included in the model.

Significance will be tested at the 2-sided 5% level using Satterthwaite’s degrees of freedom method. The lmer/lmerTest package in R will be used for mixed effect modelling. Summary statistics will include means and standard deviations (for each Group x Event, beta estimate, 95% confidence interval and p value). Residuals of the linear mixed effect model will be checked for normality.

# Safety Analyses

## Adverse Events

Adverse events will be determined using the GASE and Table 3 populated. A similar Table restricted to AEs occurring that a participant attributes to the intervention will also be constructed. This analysis will be purely descriptive.

Table 3: Template Adverse Event Table for NALDEP (severe column missing)

| Symptom | Placebo  *n participants* | LDN  *n participants* | Event Severity | | | |
| --- | --- | --- | --- | --- | --- | --- |
|  |  |  | Mild  *n participants* | | Moderate  *n participants* | |
|  |  |  | Placebo | LDN | Placebo | LDN |
| *Gastrointestinal disorders* |  |  |  |  |  |  |
| Gastrointestinal discomfort | x | x | x | x | x | x |
| *General system disorders* |  |  |  |  |  |  |
| Chest pain | x | x | x | x | x | x |
| *Total* | *x* | x | x | x | x | x |

## Deaths, Serious Adverse Events and other Significant Adverse Events

Any Deaths and Serious Adverse Events will be listed and described on a case-by-case basis.

## Pregnancies

Any pregnancies and their outcomes will be listed and described on a case-by-case basis.

# Other Analyses

The analysis described in this SAP cover only those for the primary and safety endpoints described in the Protocol. Analyses of secondary and tertiary/exploratory objectives are not included here and will be analyzed subsequently, and in the case of tertiary/exploratory objectives will be considered exploratory/hypotheses generating analyses

# Reporting Conventions

All numerical values and p-values ≥0.001 will be reported to 3 decimal places; p-values less than 0.001 will be reported as “<0.001”.

# Quality Assurance of Statistical Programming

Following locking of the Redcap database, data will be exported into format for direct input into R/RStudio statistical Software. All statistical analyses described in this document will be performed in R/RStudio software. All analyses will use code written by an investigator.

Statistical outputs will be into RMarkdown files which will include:

- date and time of analyses
- the name of the code file that produced the analysis
- the author

At the start of any code file there will be a set of comments that give

- the author
- the date and time of writing
- references to inputs and outputs

# Summary of Changes to the Protocol and/or SAP

Not applicable to this version.

# References

American Psychiatric Association. (2013). Diagnostic and statistical manual of mental disorders (5th ed.). <https://doi.org/10.1176/appi.books.9780890425596>

Bullmore, E. (2018). *The inflamed mind: a radical new approach to depression*. Picador.

Chamberlain, S. R., Cavanagh, J., de Boer, P., Mondelli, V., Jones, D. N. C., Drevets, W. C., Cowen, P. J., Harrison, N. A., Pointon, L., Pariante, C. M., & Bullmore, E. T. (2019). Treatment-resistant depression and peripheral C-reactive protein. *British Journal of Psychiatry*, *214*(1), 11-19. <https://doi.org/10.1192/bjp.2018.66>

Hindmarch, I. (2001). Expanding the horizons of depression: beyond the monoamine hypothesis. *Human Psychopharmacology*, *16*(3), 203-218. <https://doi.org/10.1002/hup.288>

J, T., L, B., T, H., J, R., M, W., & M, H. (2018). Different ways to estimate treatment effects in randomised controlled trials. *Contemp Clin Trials Commun*, *10*, 80-85. <https://doi.org/10.1016/j.conctc.2018.03.008>

Mischoulon, D., Hylek, L., Yeung, A. S., Clain, A. J., Baer, L., Cusin, C., Ionescu, D. F., Alpert, J. E., Soskin, D. P., & Fava, M. (2017). Randomized, proof-of-concept trial of low dose naltrexone for patients with breakthrough symptoms of major depressive disorder on antidepressants. *Journal of Affective Disorders*, *208*, 6-14. <https://doi.org/10.1016/j.jad.2016.08.029>

Raison, C. L., Capuron, L., & Miller, A. H. (2006). Cytokines sing the blues: inflammation and the pathogenesis of depression. *Trends in Immunology*, *27*(1), 24-31. <https://doi.org/10.1016/j.it.2005.11.006>

Rief, W., Barsky, A. J., Glombiewski, J. A., Nestoriuc, Y., Glaesmer, H., & Braehler, E. (2011). Assessing general side effects in clinical trials: reference data from the general population. *pharmacoepidemiology and drug safety*, *20*(4), 405-415. <https://doi.org/10.1002/pds.2067>

Thase, M. E., & Rush, A. J. (1997). When at first you don't succeed: sequential strategies for antidepressant nonresponders. *Journal of Clinical Psychiatry*, *58*(Suppl 13), 23-29.

The Royal Australian and New Zealand College of Psychiatrists. (2016). *The economic cost of serious mental illness and comorbidities in Australia and New Zealand*. <https://www.ranzcp.org/Files/Publications/RANZCP-Serious-Mental-Illness.aspx>

World Health Organisation. *Depression*. <https://www.who.int/en/news-room/fact-sheets/detail/depression>: Accessed 14 May 2024

# Listing of Tables, Listings and Figures

[Figure 1: Flowchart of participant stratification based on level of high sensitivity C-reactive protein (hsCRP). 7](#_Toc176265000)

[Figure 2: Standard Protocol Items: Recommendations for Interventional Trials (SPIRIT) 10](#_Toc176265001)

[Figure 3: Blank CONSORT diagram for NALDEP 14](#_Toc176265002)

[Table 1: Minimum demographic table from NALDEP 15](#_Toc176265017)

[Table 2 Treatment compliance summary table for NALDEP 16](#_Toc176265018)

[Table 3: Template Adverse Event Table for NALDEP (severe column missing) 17](#_Toc176265019)
