# Supplementary material for: Low-dose naltrexone as an adjunctive treatment for major depressive disorder: findings from a randomized, double-blind, placebo-controlled hybrid parallel-arm study
Source: Front Pharmacol. 2026 Mar 6;17:1767654. doi: 10.3389/fphar.2026.1767654 (PMC13002618; doi:10.3389/fphar.2026.1767654)
Supplement: Supplementary file 1 [file Supplementaryfile1.zip › Supplementary Results.DOCX]

Supplementary Material

# Supplementary Tables

Table S1: Reasons for screening failures

| Reason | *n* (%) |
| --- | --- |
| Medical History | 1 (1.9) |
| Psychological evaluation | 3 (5.7) |
| MADRS score < 18 | 14 (26.4) |
| hs-CRP > 1mg/L and < 3mg/L | 14 (26.4) |
| hs-CRP ≤ 1 mg/L but stratum recruitment closed | 21 (39.6) |
| *Total* | 53 |

Table S2: Study intervention compliance by treatment Group

| Doses taken (%) | Placebo N (%) | LDN N (%) |
| --- | --- | --- |
| ≥75% (based on returned capsule count) | 15 (83) | 19 (100) |
| <75% (based on returned capsule count) | 0 | 0 |
| ≥75% (based on self-reported data) | 3 (17) | 0 |
| <75% (based on self-reported data) | 0 | 0 |

Table S3: Number of participants self-reporting adverse events during the double-blind placebo-controlled phase

|  | | | Event severity | | | | | |
| --- | --- | --- | --- | --- | --- | --- | --- | --- |
|  |  |  | Mild *n* participants | | Moderate *n* participants | | Severe *n* participants | |
| Event | Placebo *n* participants | LDN  *n* participants | Placebo | LDN | Placebo | LDN | Placebo | LDN |
|  |  |  | *n* | *n* | *n* | *n* | *n* | *n* |
| *Gastrointestinal disorders* |  |  |  |  |  |  |  |  |
| Abdominal pain | 6 | 6 | 3 | 4 | 2 | 1 | 1 | 1 |
| Constipation | 7 | 5 | 6 | 4 | 1 | 1 | 0 | 0 |
| Diarrhea | 6 | 6 | 5 | 3 | 1 | 3 | 0 | 0 |
| Dry mouth | 8 | 8 | 5 | 5 | 2 | 3 | 1 | 0 |
| Increased appetite | 6 | 6 | 4 | 5 | 2 | 1 | 0 | 0 |
| Nausea | 8 | 14 | 6 | 12 | 2 | 2 | 0 | 0 |
| Reduced appetite | 8 | 9 | 4 | 6 | 4 | 3 | 0 | 0 |
| *General disorders and administration site conditions* |  |  |  |  |  |  |  |  |
| Chest pain | 0 | 4 | 0 | 3 | 0 | 1 | 0 | 0 |
| Fever, increased temperature | 3 | 2 | 3 | 2 | 0 | 0 | 0 | 0 |
| *Musculoskeletal and connective tissue disorders* |  |  |  |  |  |  |  |  |
| Back pain | 12 | 7 | 7 | 4 | 4 | 3 | 1 | 0 |
| Joint pain | 9 | 5 | 7 | 5 | 2 | 0 | 0 | 0 |
| Muscle pain | 11 | 7 | 6 | 3 | 5 | 4 | 0 | 0 |
| *Nervous system disorders* |  |  |  |  |  |  |  |  |
| Dizziness | 7 | 9 | 7 | 7 | 0 | 2 | 0 | 0 |
| Fatigue, loss of energy | 12 | 11 | 3 | 3 | 9 | 6 | 0 | 2 |
| Headache | 13 | 15 | 8 | 8 | 3 | 4 | 2 | 3 |
| Tremor | 4 | 4 | 3 | 3 | 1 | 1 | 0 | 0 |
| *Psychiatric disorders* |  |  |  |  |  |  |  |  |
| Nightmare, or abnormal dreams | 8 | 13 | 5 | 8 | 2 | 4 | 1 | 1 |
| Agitation | 11 | 7 | 7 | 6 | 4 | 1 | 0 | 0 |
| Anxiety, fearfulness | 15 | 14 | 7 | 9 | 8 | 4 | 0 | 1 |
| Depressed mood | 15 | 17 | 6 | 7 | 9 | 8 | 0 | 2 |
| Irritability, nervousness | 10 | 10 | 4 | 8 | 6 | 1 | 0 | 1 |
| Insomnia, sleeping problems | 14 | 15 | 11 | 11 | 3 | 4 | 0 | 0 |
| *Reproductive system and breast disorders* |  |  |  |  |  |  |  |  |
| Painful or irregular menstruation | 3 | 4 | 1 | 2 | 0 | 2 | 2 | 0 |
| Problems with sexual performance or sex organs | 4 | 2 | 2 | 1 | 2 | 1 | 0 | 0 |
| *Respiratory, thoracic and mediastinal disorders* |  |  |  |  |  |  |  |  |
| Breathing problems | 3 | 2 | 3 | 2 | 0 | 0 | 0 | 0 |
| *Skin and subcutaneous tissue disorders* |  |  |  |  |  |  |  |  |
| Tendency to develop bruises | 7 | 3 | 5 | 2 | 2 | 1 | 0 | 0 |
| Hair loss | 3 | 2 | 3 | 2 | 0 | 0 | 0 | 0 |
| Skin rash or itching | 6 | 5 | 5 | 4 | 1 | 1 | 0 | 0 |
| *Vascular disorders* |  |  |  |  |  |  |  |  |
| Hot flashes | 1 | 4 | 1 | 4 | 0 | 0 | 0 | 0 |
| Low blood pressure | 3 | 1 | 1 | 1 | 2 | 0 | 0 | 0 |
| Palpitations, irregular heartbeat | 3 | 5 | 3 | 5 | 0 | 0 | 0 | 0 |
| Abnormal sweating | 6 | 4 | 4 | 2 | 2 | 2 | 0 | 0 |
| Events were recorded via the GASE questionnaire at weeks 1, 2, 4, 8, and 12. Severity reflects the highest rating reported between baseline and week 12. Events reported by ≥15% of participants in either group are shown.  This table includes all self-reported adverse events irrespective of whether participants believed they were related or unrelated to the intervention | | | | | | | | |

Table S4: Additional safety data detailing the number of participants self-reporting adverse effects, including those effects self-reported to be related to the intervention, and including all participants who received blinded or open-label LDN (*n* = 35).

|  | | | Event severity *n* participants | | | | | |
| --- | --- | --- | --- | --- | --- | --- | --- | --- |
| Event | *n* participants | Related *n* participants | Mild | Mild (related) | Moderate | Moderate (related) | Severe | Severe (related) |
| *Gastrointestinal disorders* |  |  |  |  |  |  |  |  |
| Abdominal pain | 14 | 3 | 8 | 1 | 4 | 1 | 2 | 1 |
| Constipation | 11 | 3 | 7 | 2 | 4 | 1 | 0 | 0 |
| Diarrhea | 13 | 2 | 8 | 1 | 5 | 1 | 0 | 0 |
| Dry mouth | 19 | 10 | 12 | 5 | 6 | 4 | 1 | 1 |
| Increased appetite | 15 | 6 | 12 | 5 | 3 | 1 | 0 | 0 |
| Nausea | 22 | 9 | 13 | 6 | 8 | 3 | 1 | 0 |
| Reduced appetite | 18 | 5 | 14 | 4 | 4 | 1 | 0 | 0 |
| Vomiting | 6 | 2 | 5 | 2 | 0 | 0 | 1 | 0 |
| *General disorders and administration site conditions* |  |  |  |  |  |  |  |  |
| Chest pain | 6 | 2 | 4 | 1 | 2 | 1 | 0 | 0 |
| *Musculoskeletal and connective tissue disorders* |  |  |  |  |  |  |  |  |
| Back pain | 20 | 3 | 14 | 2 | 5 | 1 | 1 | 0 |
| Joint pain | 13 | 3 | 10 | 3 | 3 | 0 | 0 | 0 |
| Muscle pain | 17 | 4 | 8 | 2 | 9 | 2 | 0 | 0 |
| *Nervous system disorders* |  |  |  |  |  |  |  |  |
| Dizziness | 14 | 5 | 11 | 2 | 2 | 2 | 1 | 1 |
| Fatigue, loss of energy | 24 | 7 | 5 | 4 | 13 | 1 | 6 | 2 |
| Headache | 26 | 12 | 14 | 7 | 9 | 3 | 3 | 2 |
| Tremor | 7 | 3 | 6 | 3 | 1 | 0 | 0 | 0 |
| *Psychiatric disorders* |  |  |  |  |  |  |  |  |
| Nightmare, or abnormal dreams | 21 | 12 | 11 | 8 | 8 | 2 | 2 | 2 |
| Agitation | 16 | 5 | 12 | 3 | 4 | 2 | 0 | 0 |
| Anxiety, fearfulness | 28 | 6 | 17 | 4 | 8 | 1 | 3 | 1 |
| Depressed mood | 30 | 6 | 13 | 2 | 14 | 3 | 3 | 1 |
| Irritability, nervousness | 21 | 6 | 12 | 3 | 7 | 2 | 2 | 1 |
| Insomnia, sleeping problems | 24 | 12 | 14 | 9 | 10 | 3 | 0 | 0 |
| *Reproductive system and breast disorders* |  |  |  |  |  |  |  |  |
| Painful or irregular menstruation | 9 | 1 | 4 | 0 | 3 | 0 | 2 | 1 |
| *Skin and subcutaneous tissue disorders* |  |  |  |  |  |  |  |  |
| Tendency to develop bruises | 10 | 5 | 8 | 4 | 2 | 1 | 0 | 0 |
| Hair loss | 6 | 1 | 6 | 1 | 0 | 0 | 0 | 0 |
| Skin rash or itching | 11 | 2 | 8 | 1 | 3 | 1 | 0 | 0 |
| *Vascular disorders* |  |  |  |  |  |  |  |  |
| Hot flashes | 5 | 2 | 5 | 2 | 0 | 0 | 0 | 0 |
| Palpitations, irregular heartbeat | 7 | 3 | 7 | 3 | 0 | 0 | 0 | 0 |
| Abnormal sweating | 7 | 3 | 2 | 1 | 5 | 2 | 0 | 0 |
| Events were recorded via the GASE questionnaire at weeks 1, 2, 4, 8, 12, 13, 14, 20 and 24. This table includes all self-reported adverse events irrespective of whether participants believed they were related or unrelated to the intervention. Severity reflects the highest rating reported between baseline and week 24. | | | | | | | | |

Table S5: Deblinding and expectancy parameters by treatment group

|  | Placebo (*n* = 18) | LDN (*n =* 19) | *t, p* |
| --- | --- | --- | --- |
| Participant correct guess^1^ *n* (%) | 8 (50%) | 5 (38%) | NA |
| Stanford Expectation of Treatments Scale *M* (*sd*) |  |  |  |
| Positive expectancy score | 3.6 (0.9) | 3.7 (1.5) | 0.25, 0.80 |
| Negative expectancy score | 2.9 (1.8) | 2.5 (1.7) | -1.04, 0.30 |
| ^1^ Denominator for percentage calculations were LDN *n =* 13 and placebo *n =* 16 due to dropouts  Note: SETs data were compared between groups using *t-*tests | | |  |

Table S6: Mean differences (LDN-placebo) in the change in MADRS scores between treatment groups at each time point adjusted for baseline, age, sex and log(baseline hsCRP)

| Timepoint | Adjusted mean difference | 95% CI | p value |
| --- | --- | --- | --- |
| 2 weeks | -1.783 | -5.916, 2.350 | 0.399 |
| 4 weeks | 2.004 | -2.186, 6.195 | 0.350 |
| 8 weeks | 4.126 | -0.182, 8.434 | 0.062 |
| 12 weeks | -0.217 | -4.610, 4.176 | 0.923 |
| Note: The linear mixed model included timepoint dummy variables, interaction terms between timepoint and treatment group, age, sex and log(baseline hsCRP) as fixed effects, and participants as a random effect. All model assumptions were checked using Q-Q and residuals versus fitted values plots. | | | |

Table S7: Descriptive statistics for secondary and exploratory outcome measures and mean differences (LDN-placebo) in the change in each outcome measure between treatment groups at 12 weeks adjusted for baseline.

| Outcome | Time | Placebo | | | LDN | | | Adjusted mean difference | 95% CI | *p* |
| --- | --- | --- | --- | --- | --- | --- | --- | --- | --- | --- |
|  |  | *n* | M (sd) | Range (min-max) | *n* | M (sd) | Range (min-max) |  |  |  |
| Log(hsCRP) | Baseline | 18 | 0.051 (0.696) | -1.602 - 1.144 | 19 | 0.073 (0.859) | -1.125 - 1.597 |  |  |  |
|  | 12 weeks | 15 | 0.234 (0.900) | -1.602 - 1.805 | 13 | 0.197 (0.802) | -1.602 - 1.622 | -0.225 | -0.585, 0.136 | 0.231 |
| BDI - II | Baseline | 18 | 24.944 (8.954) | 10 - 38 | 19 | 29.842 (10.590) | 6 - 44 |  |  |  |
|  | 12 weeks | 16 | 10.813 (9.086) | 1 - 35 | 13 | 12.462 (10.453) | 2 - 40 | -0.755 | -6.353, 4.844 | 0.792 |
| BADS total | Baseline | 18 | 72.667 (16.971) | 49 - 116 | 19 | 64.053 (27.381) | 12 - 110 |  |  |  |
|  | 12 weeks | 16 | 95.750 (15.554) | 64 - 120 | 13 | 96.923 (28.646) | 52 - 134 | 5.579 | -8.232, 19.425 | 0.429 |
| BADS: AC | Baseline | 18 | 16.278 (6.163) | 7 - 29 | 19 | 14.105 (7.781) | 3 - 32 |  |  |  |
|  | 12 weeks | 16 | 20.313 (6.030) | 11 - 32 | 13 | 21.538 (9.333) | 7 - 40 | 1.990 | -2.667, 6.647 | 0.404 |
| BADS: AR | Baseline | 18 | 22.389 (8.507) | 3 - 35 | 19 | 18.737 (10.088) | 0 - 36 |  |  |  |
|  | 12 weeks | 16 | 33.313 (7.726) | 2 - 45 | 13 | 33.385 (10.096) | 16 - 47 | 2.416 | -3.013, 7.844 | 0.385 |
| BADS: WS | Baseline | 18 | 14.778 (5.897) | 5 - 29 | 19 | 12.737 (6.624) | 1 - 25 |  |  |  |
|  | 12 weeks | 16 | 18.063 (6.159) | 6 - 27 | 13 | 19.385 (5.546) | 13 - 30 | 2.154 | -1.902, 6.210 | 0.300 |
| BADS: SI | Baseline | 18 | 19.222 (5.331) | 8 - 28 | 19 | 18.474 (8.572) | 0 - 29 |  |  |  |
|  | 12 weeks | 16 | 24.063 (4.479) | 17 - 30 | 13 | 22.615 (8.392) | 9 - 30 | -1.261 | -5.313, 2.790 | 0.543 |
| SF - 36 General Health | Baseline | 18 | 53.611 (18.535) | 30 - 90 | 19 | 53.947 (19.691) | 15 - 80 |  |  |  |
|  | 12 weeks | 16 | 65.625 (18.246) | 30 - 95 | 13 | 65.769 (19.879) | 15 - 85 | -1.209 | -10.051, 7.633 | 0.790 |
| SF - 36 Health Change | Baseline | 18 | 52.778 (18.960) | 25 - 100 | 19 | 56.579 (23.336) | 25 - 100 |  |  |  |
|  | 12 weeks | 16 | 64.063 (22.302) | 50 - 100 | 13 | 61.538 (19.406) | 50 - 100 | -3.517 | -17.806, 10.772 | 0.632 |
| SF - 36 Mental Health | Baseline | 18 | 42.667 (14.324) | 16 - 68 | 19 | 42.526 (15.215) | 20 - 72 |  |  |  |
|  | 12 weeks | 16 | 61.250 (15.438) | 28 - 84 | 13 | 60.308 (19.559) | 20 - 80 | -1.905 | -11.814, 8.004 | 0.708 |
| SF - 36 Pain | Baseline | 18 | 69.028 (17.408) | 35 - 100 | 19 | 71.447 (22.659) | 22.5 - 100 |  |  |  |
|  | 12 weeks | 16 | 73.438 (23.994) | 22.5 - 100 | 13 | 78.462 (13.289) | 57.5 - 100 | 5.599 | -7.118, 18.316 | 0.392 |
| SF - 36 Physical Function | Baseline | 18 | 86.944 (17.751) | 45 - 100 | 19 | 85.789 (19.095) | 45 - 100 |  |  |  |
|  | 12 weeks | 16 | 89.688 (16.780) | 45 - 100 | 13 | 89.615 (10.890) | 65 - 100 | 0.048 | -8.466, 8.562 | 0.991 |
| SF - 36 Role Emotional | Baseline | 18 | 18.519 (30.726) | 0 - 100 | 19 | 12.281 (19.909) | 66.667 - 100 |  |  |  |
|  | 12 weeks | 16 | 52.083 (40.311) | 0 - 100 | 13 | 53.846 (39.764) | 0 - 100 | 4.536 | -17.744, 26.816 | 0.691 |
| SF - 36 Role Physical | Baseline | 18 | 63.889 (41.322) | 0 - 100 | 19 | 71.053 (35.613) | 0 - 100 |  |  |  |
|  | 12 weeks | 16 | 70.313 (40.020) | 0 - 100 | 13 | 92.308 (15.761) | 50 - 100 | 22.707 | -0.813, 46.226 | 0.064 |
| SF - 36 Social Function | Baseline | 18 | 61.806 (20.325) | 25 - 100 | 19 | 44.079 (23.706) | 0 - 75 |  |  |  |
|  | 12 weeks | 16 | 69.531 (17.059) | 25 - 87.5 | 13 | 66.346 (28.129) | 0 - 100 | 11.578 | -0.224, 23.379 | 0.062 |
| SF - 36 Vitality | Baseline | 18 | 27.500 (13.089) | 5 - 55 | 19 | 17.368 (13.475) | 0 - 45 |  |  |  |
|  | 12 weeks | 16 | 40.938 (16.250) | 15 - 70 | 13 | 36.154 (18.836) | 10 - 75 | -0.059 | -10.051, 9.933 | 0.991 |
| SicknessQ | Baseline | 18 | 12.111 (4.801) | 4 - 20 | 19 | 13.000 (6.523) | 2 - 26 |  |  |  |
|  | 12 weeks | 16 | 8.688 (5.003) | 1 - 16 | 13 | 8.000 (5.260) | 1 - 18 | -0.994 | -4.511, 2.523 | 0.582 |
| POMS Anger | Baseline | 18 | 3.944 (7.892) | 0 - 28 | 19 | 4.842 (6.167) | 0 - 18 |  |  |  |
|  | 12 weeks | 16 | 2.750 (4.041) | 0 - 13 | 13 | 3.077 (6.075) | 0 - 21 | 0.187 | -4.058, 4.432 | 0.931 |
| POMS Confusion | Baseline | 18 | 9.667 (3.106) | 3 - 17 | 19 | 9.579 (4.363) | 2 - 17 |  |  |  |
|  | 12 weeks | 16 | 7.063 (3.803) | 3 - 19 | 13 | 6.462 (3.455) | 2 - 15 | -0.253 | -2.692, 2.187 | 0.840 |
| POMS Depression | Baseline | 18 | 10.833 (8.867) | 0 - 36 | 19 | 14.526 (12.080) | 1 - 36 |  |  |  |
|  | 12 weeks | 16 | 6.438 (9.345) | 0 - 37 | 13 | 7.769 (10.084) | 0 - 28 | -0.729 | -5.980, 4.522 | 0.787 |
| POMS Fatigue | Baseline | 18 | 11.056 (4.608) | 4 - 19 | 19 | 11.632 (5.610) | 4 - 21 |  |  |  |
|  | 12 weeks | 16 | 8.750 (5.471) | 0 - 17 | 13 | 8.923 (6.487) | 0 - 19 | 0.203 | -3.385, 3.790 | 0.912 |
| POMS total | Baseline | 18 | 37.833 (27.577) | 3 - 107 | 19 | 44.316 (30.840) | -2 - 109 |  |  |  |
|  | 12 weeks | 16 | 22.375 (27.594) | -10 - 101 | 13 | 25.154 (33.269) | -6 - 98 | 1.261 | -16.742, 19.264 | 0.891 |
| POMS Tension | Baseline | 18 | 11.778 (8.349) | 2 - 26 | 19 | 12.316 (6.369) | 1 - 26 |  |  |  |
|  | 12 weeks | 16 | 9.750 (7.000) | 1 - 25 | 13 | 8.154 (7.647) | 1 - 24 | -1.391 | -6.305, 3.524 | 0.581 |
| POMS Vigor | Baseline | 18 | 9.444 (4.718) | 3 - 21 | 19 | 8.579 (4.439) | 3 - 18 |  |  |  |
|  | 12 weeks | 16 | 12.375 (5.548) | 5 - 22 | 13 | 9.231 (4.362) | 1 - 16 | 2.714 | -5.673, 0.244 | 0.079 |
| Note: Linear mixed models were adjusted for baseline and included timepoint dummy variables and interaction terms between timepoint and treatment group as fixed effects, and participants as a random effect. All model assumptions were checked using Q - Q and residuals versus fitted values plots.  hsCRP = high sensitivity C - reactive protein, BDI - II = Beck Depression Inventory II, BADS = Behavioural Activation in Depression Scale, AC = activation subscale, AR = avoidance/rumination subscale, WS = work/school impairment subscale, SI = social impairment subscale, SF - 36 = Short Form 36, SicknessQ = Sickness Questionnaire, POMS = Profile of Mood States | | | | | | | | | | |
